# Bee Collected Pollen with Enhanced Health Benefits, Produced by Fermentation with a Kombucha Consortium

**DOI:** 10.3390/nu10101365

**Published:** 2018-09-23

**Authors:** Elena Uțoiu, Florentina Matei, Agnes Toma, Camelia Filofteia Diguță, Laura Mihaela Ștefan, Sorin Mănoiu, Virgil Valeriu Vrăjmașu, Ionuț Moraru, Anca Oancea, Florentina Israel-Roming, Călina Petruța Cornea, Diana Constantinescu-Aruxandei, Angela Moraru, Florin Oancea

**Affiliations:** 1 Faculty of Biotechnologies, University of Agronomic Sciences and Veterinary Medicine of Bucharest, Bucharest 011464, Romania; Elena.Utoiu@gmail.com (E.U.); Florentina.Matei@biotehnologii.usamv.ro (F.M.); camifilo@yahoo.com (C.F.D.); VirgilVrajamasu@yahoo.com (V.V.V.); florentinarom@yahoo.com (F.I.-R.); pccornea@yahoo.com (C.P.C.); Angela.Moraru@medicagroup.ro (A.M.); 2 Department of Cellular and Molecular Biology, National Institute of Research and Development for Biological Sciences, Bucharest 060031, Romania; agnes12ro@yahoo.com.au (A.T.); LauraMihaelaStefan@yahoo.com (L.M.S.); spagiricus@yahoo.com (S.M.); Oancea.Anca@gmail.com (A.O.); 3 Medica Laboratories Srl, Otopeni 075100, Romania; IonutMoraru@pro-natura.ro; 4 Departments of Biotechnology and Bioresources, National Institute for Research & Development in Chemistry and Petrochemistry-ICECHIM, Bucharest 060021, Romania; doro31@gmail.com

**Keywords:** pollen, multi-floral, bee collected, fermentation, symbiotic culture of bacteria and yeasts—SCOBY, lactic acid bacteria—LAB, anti-oxidant polyphenols, soluble silicon, short-chain fatty acids—SCFA

## Abstract

The bioavailability of pollen bioactive compounds for humans is limited. In this study, our aim was to enhance the health-related benefits of pollen by fermentation with a Kombucha/SCOBY (symbiotic culture of bacteria and yeasts) consortium. We performed the fermentation of pollen suspended from the beginning with SCOBY on sweetened green tea or on Kombucha vinegar, by adding pollen after 20 days of Kombucha fermentation. We analyzed: formation of bioactive compounds (anti-oxidant polyphenols, soluble silicon, hydroxy-acids, short chain fatty acids—SCFA); parameters related to Kombucha fermentation (dynamics of lactic acid bacteria—LAB, formation of organic acids, soluble sugar evolution on Kombucha vinegar); the influence of Kombucha fermentation on pollen morphology and ultrastructure; in vitro cytotoxic and antitumoral effects of the Kombucha fermented pollen. The pollen addition increases LAB proportion in the total number of SCOBY microbial strains. SEM images highlight the adhesion of the SCOBY bacteria to pollen. Ultrastructural analysis reveals the release of the pollen content. The content of bioactive compounds (polyphenols, soluble silicon species and SCFA) is higher in the fermented pollen and the product shows a moderate antitumoral effect on Caco-2 cells. The health benefits of pollen are enhanced by fermentation with a Kombucha consortium.

## 1. Introduction

Bee collected pollen is a “superfood”, a nutraceutical with high biological value ingredients, e.g., essential amino acids, (pro)vitamins, essential fatty acids, minerals and anti-oxidant polyphenols [1]. Due to such phytonutrients with high biological value, commercial mixtures of multi-floral, bee collected pollen, have been proven to have anti-microbial, antimutagenic, antioxidant and anti-inflammatory effects [2]. Antitumoral, immuno-modulatory and anti-metabolic syndrome (cardio-protective, anti-hypertensive, anti-atherosclerotic and anti-diabetic) activities were also reported for bee (collected) pollen [3]. Recently, a modulation effect exerted by carbohydrates from bee pollen on gut microbiota was suggested [4]. However, the bioavailability of phytonutrients from bee pollen is significantly limited by the complex structure of the pollen grain wall and the recalcitrance to biodegradation of the rigid pollen outer wall, exine [5]. The exine resistance results from its major component, sporopollenin, a biopolymer with extremely high stability and very high resistance to (bio)degradation, including to the action of digestive enzymes [6]. Only the animals which developed mechanisms for phytonutrients extraction from pollen grains (e.g., mechanical crack-up, pseudogermination/fake germination, osmotic shock, outer wall piercing sharp mouth parts) are able to use more than 50% of the pollen grain content [7]. Humans have not developed such mechanisms.

Various mechanical, chemical and biotechnological processes have been proposed to enhance the bioavailability of phytonutrients from bee collected pollen. A non-exhaustive list includes: high-shearing [8], thermal shocks, alone or combined with enzymatic treatment [5], wet milling [9], ultrasonication [10] combined with high-shearing [11] or enzymatic treatment [12], chemical extraction of polyphenols with supercritical fluid [13], fermentation [14,15,16,17,18].

Pollen fermentation is similar to the naturally evolved processes, targeted on enhancement of pollen nutritional value. In the bee hive, pollen is fermented and stored with honey, into wax sealed honey combs. The resulting products, the bee bread, combine the pollen phytonutrients (with increased bioavailability) and bioactive compounds formed during pollen fermentation [19]. As a result of such a combination, bee bread has a higher content in essential amino acids and vitamins (e.g., from B and K groups) and more bioactive polyphenols [20]. Lactic acid bacteria [21], yeasts [22] and endospore forming gram negative bacteria [23] are involved into pollen fermentation and bee brood formation.

Biotechnological alternatives to bee bread have been proposed. The bee bread is not produced in large quantities and it is difficult to harvest from the honey combs. Several processes of (semi)solid pollen fermentation were developed in an attempt to produce products similar to bee bread [20]. Enhancement of antioxidant polyphenols in fermented pollen was previously reported [5,14]. The addition of pollen to products resultant from lactic acid bacteria and/or yeasts fermentation has been also tested. One of the main results of such biotechnological processes was a better quality of the fermented products. Better white wines were produced after inclusion of pollen into white grape must [24]. Pollen addition also increases the quality of the yogurt [25] and of the other milk fermented beverages [25].

Our aim was to obtain a complex product, which includes various health-related compounds, by fermentation of the bee collected pollen with a Kombucha consortium, a symbiotic colony of bacteria and yeast (SCOBY). Kombucha is an oriental traditional beverage, produced from sweetened green or black tea by fermentation with a symbiotic consortium of bacteria and yeast (SCOBY). The consortium, including anaerobic and aerobic microbial strains, is embedded within a cellulose membrane, which floats on the fermented tea beverage (the soup) and which is a good source of bacterial nanocellulose [26]. The SCOBY fermentation process is static and the usual fermentation time is from 7 up to 30 days at room temperature [27]. Consumption of Kombucha is associated with various health benefits: protection against various pathologies induced by the radical oxygen species (ROS); detoxifying activities, mainly because of the accumulation of organic acids (acetic, gluconic. glucuronic, lactic); antimicrobial effects, resultant from polyphenols and antibiotics/bacteriocins; increased immunity, because of both antioxidant activity and bioactive ingredients, such as B and C vitamins [28,29,30]. Generally, the SCOBY consortium is dominated by acetic bacteria like *Gluconoacetobacter* sp. and yeast like *Zygosaccharomyces* or *Dekkera* [31,32,33]. Lactic acid bacteria (LAB) have been reported in both Kombucha layers, soup and pellicle, counting up to 30% of the SCOBY microbial cells [32]. Different LAB species have been identified in SCOBY, such as *Lactobacillus* sp., *Lactococcus* sp., *Leuconostoc* sp. or *Pediococcus pentosaceus* [32,33,34]. However, very few reports refer to LAB from SCOBY as potential probiotics [33,35]. To the best of our knowledge, there are no studies related to postbiotic compounds produced by LAB fermentation from SCOBY. Postbiotics are soluble compounds from probiotics, i.e., microbial metabolites, including short chain fatty acids such as acetic, propionic and butyric acids, and microbial components, such as (lipo)teichoic acids, peptidoglycans/mural peptides, cell surface proteins, with potential significant health benefits [36,37].

To demonstrate the enhancement of health benefits on pollen fermented by the Kombucha consortium, we analyzed the following: the formation of bioactive compounds (anti-oxidant polyphenols, soluble silicon, postbiotic short-chain fatty acids); parameters related to SCOBY/Kombucha fermentation (dynamics of LAB in SCOBY, formation of specific organic acids, evolution of soluble sugars in Kombucha vinegar); the influence of Kombucha fermentation on pollen morphology and ultrastructure; in vitro cytotoxic and antitumoral effects of the resultant Kombucha fermented pollen. Our focus was not only on anti-oxidant polyphenols, as such compounds have already been demonstrated to be enhanced during pollen fermentation [5,14,19]. We were also interested in biosilica solubilization and post-biotic short chain fatty acids (SCFA). Silicon is one of the main components of the pollen cell wall [38]. In the plant cell wall, silicon is present as biosilica, SiO_2_·*n*H_2_O, which is formed after orthosilicic acid polycondensation and precipitation [39]. Biosilica increases plant cell wall resistance to (bio)degradation [40]. Its solubilization weakens the pollen structure and provides an additional health-related benefit, due to the formation of soluble silicon species. These soluble silicon species, especially orthosilicic acid, H_4_SiO_4_, have been proven to exert several beneficial effects on humans, such as maintenance of bone health [41], including osteoporosis prevention [42,43], or optimal connective tissue function, stimulation of the immune system and Alzheimer’s disease prevention [44,45]. Post-biotic short chains fatty acids (e.g., acetic, propionic, butyric acids, produced by the prebiotic strains) are important for proper colonic function, protection against colorectal cancer and modulation of intestinal immune, inflammatory and metabolic functions [37].

## 2. Materials and Methods

### 2.1. Biological Material

Samples of bee pollen were harvested from a pollen trap mounted in the beehives from the Bucharest-Ilfov area, in late spring to early summer of 2018. The samples were removed weekly and stored in a freezer (−18 °C). The composition of the multiflora bee collected pollen was microscopically evaluated, by using the unacetolysed method [46], with a total magnification (*x*40). For ultrastructural evaluation on SEM mono-floral pollen (apple pollen), more uniform in shape was used. The identifications were done by comparison to references from European pollen atlases [47,48]. The SCOBY/Kombucha consortium contained: lactic acid bacteria, acetobacteria, from two main genera, *Gluconoacetobacter* and *Komagataeibacter*, and yeasts from several genera, including *Brettanomyces*/*Dekkera*, *Zygosaccharomyces* and *Pichia* [26]. For the in vitro evaluation of the Kombucha fermented pollen, normal mouse fibroblast cell line, NCTC clone L929 (for biocompatibility), and two transformed human cell lines, laryngeal epidermoid carcinoma cells (Hep-2) and human colon adenocarcinoma cells (Caco-2) (for antitumor activity determination) were used. All cell lines were purchased from the European Collection of Cell Culture (ECACC).

### 2.2. Pollen Fermentation with the Kombucha Consortium

For Kombucha/SCOBY fermentation the tea infusion was produced from 5 g of green tea (Basilur green tea, Ceylon) infused for 15 min into 1 L of boiling sterile water. The non-extracted material was removed by filtration on Whatman no. 5 filter paper. The resultant tea infusion was sweetened with 70 g/L of sucrose (commercial crystalline sugar). For laboratory level investigations, 2 L of sweetened green tea infusion were aseptically distributed into a sterile brown glass bottle of 3 L. For the process scale-up larger Simax^®^ glass vessels (Kavalier, Prague, Czech Republic), of 150 L total capacity, with 100 L working volume each, were used. For the experiments related to pollen biosilica solubilization, a 2.2 L plastic bottle made from fluoropolymers, (Nalgene™ Wide-Mouth EP Tox/TCLP Teflon™ FEP Bottle, with PFA-lined Closure, Nalge Nunc—Thermo Fisher, Rochester, NY, USA) was filled with 1.5 L of sweetened green tea infusion. For the inoculation, 10% of a previously fermented Kombucha tea (without pollen) was used. For each liter of SCOBY inoculated infusion, 50 g of multi-floral, bee collected pollen was added. Pollen was added immediately after Kombucha inoculation or after 20 days of fermentation at 28 °C (pollen addition to Kombucha vinegar). The pollen fermentation process was conducted at 28 °C, in static, microaerophilic conditions, without agitation, for a period of 30 days (for the pollen added immediately after Kombucha inoculation) and for additional 10 days, for the pollen added into Kombucha vinegar. Samples were taken under axenic conditions from the Kombucha liquid phase. The concentration of reducing sugars from the fermentation of pollen with Kombucha vinegar was determined with dinitrosalicylic acid (98%, Sigma-Aldrich, Merck Group, Darmastad, Germany) [49]. On pilot scale, the floating cellulose pellicle was collected separately at the end of the fermentation period and the remaining Kombucha liquid phase with pollen was subjected to pressure homogenization on a piston homogenizer, at 1000 bars (Pony 1200, GEA Niro Soavi, Parma, Italy). The resultant suspension was spray dried (FSD Production Minor™ Spray Dryer, GEA Niro Process Engineering, Soeborg, Denmark), under mild conditions, 135–140 °C entrance temperature, 70–75 °C exit temperature, with a flow rate of 25 L of water evaporated per hour.

### 2.3. Pollen Biosilica Solubilization

The total silicon of the initial pollen, after non-oxidative alkaline sample digestion [50], was analyzed by inductively coupled plasma optical emission spectroscopy (PerkinElmer^®^ Optima™ 7000 DV, Perkin Elmer, Waltham, MA, USA). The soluble silicon (monomers and dimers of silicic acid) was determined for samples taken every two days from the Kombucha liquid phase, using the blue silicomolybdic spectrophotometric method [51], measuring the optical density at λ = 810 nm, with a standard curve of silicic acid (pro analysis—Spectroquant^®^, EMD Millipore, Merck Group), from 5 × 10^−4^ to 5 × 10^−5^ mol/L.

### 2.4. Determination of the Total Phenolic and Flavonoid Compounds

The total polyphenols were determined using the Folin–Ciocâlteu method [52], with some modifications [53]: 750 µL of Folin–Ciocâlteu reagent, 4 mL of 15% Na_2_CO_3_ and distilled water were added to 150 µL of sample, to a final volume of 15 mL; after 2 h incubation at room temperature, the absorbance at λ = 756 nm was measured. The total phenolic compounds were expressed as caffeic acid equivalents based on a calibration curve made with known concentrations of caffeic acid. The aluminum chloride colorimetric method was used for flavonoids assay [54]: 0.5 mL of sample was mixed with 1.5 mL ethanol, 0.1 mL of 1 M potassium acetate, 0.1 mL of 10% aluminum chloride, and 2.8 mL of distilled water; after 30 min incubation at room temperature the absorbance at λ = 415 nm was measured. The flavonoids content was expressed as quercetin equivalents using a calibration curve made with known concentrations of quercetin. All reagents used were analytical grade reagents, purchased from Sigma-Algrich, Merck Group.

### 2.5. Antioxidant Activity

The antioxidant activity was determined by two complementary assays: DPPH (2,2-diphenyl-1-picryl-hydrazyl-hydrate) and TEAC (Trolox equivalent antioxidant capacity). The capacity to inhibit the DPPH radical is based on the fact that the purple color of DPPH changes to yellow when the full amount of free radicals is blocked by the antioxidants The DPPH scavenging activity was measured by using the method described by Huang et al. [52]: 150 μL DPPH solution (0.25 mM) in methanol was vigorously mixed with 15 µL of sample and 90 µL of 0.1 M Tris-HCl and the resultant mixture was incubated at 37 °C for 30 min in the dark. As positive control butylated hydroxytoluene (BHT) was used. The sample absorbance (Asample) was read at λ = 520 nm against methanol blank (Ablank), using a microplate reader (Sunrise, Tecan, Männedorf, Switzerland). The DPPH Inhibition (%) was calculated using the following formula:

% Inhibition = (1 − Asample/Ablank) × 100(1)

IC50 value, i.e., the concentration necessary to inhibit 50% of the DPPH radical was calculated using linear regression, obtained by plotting concentration vs. inhibition.

The TEAC assay is based on sample ability to inhibit the ABTS (2,2′-azino-bis(3-ethylbenzothiazoline-6-sulphonic acid)) radical compared to a standard antioxidant, Trolox, used as reference. The antioxidant capacity (TEAC) was measured based on the method of Re et al. [55], with some modifications. The ABTS radical cation was generated by reacting a 7 mM 2,2′-azino-bis (3-ethyl-benzothiazoline-6-sulfonic acid) diammonium salt (ABTS) solution with 2.45 mM potassium persulfate solution (1:1, *v*/*v*). The mixture was incubated in the dark at room temperature for 16 h. The initial absorbance of ABTS radical solution was equilibrated to a value of 0.7 ± 0.02 at λ = 734 nm. Next, 1 mL of ABTS radical solution was mixed with 0.1 mL test sample and after incubation at room temperature for 6 min, the absorbance was measured at λ = 734 nm. A calibration curve of Trolox (0–250 μM) was used to convert the absorbance into the equivalent activity of Trolox per mL sample (µg Trolox/mL).

### 2.6. Dynamics of Lactic Acid Bacteria Population During Product Fermentation

The dynamics of lactic acid bacteria population was measured using two methods: targeted Q-PCR total LAB level and direct on-plate counting of viable cells.

#### 2.6.1. Real Time PCR (qPCR) Technique

*Lactobacillus acidophilus* ATCC 4356 was used as the reference strain. For DNA extractions, *L. acidophilus* ATCC 4356 was grown in 10 mL MRS (De Man, Rogosa, Sharpe) Broth (Oxoid, Thermo Scientific, Hampshire, UK), at 37 °C, for 24 h. The bacterial cells were collected by centrifugation at 4000 rpm for 10 min and a working cell suspension of 10^7^ cells/mL was used to generate the standard curve. The DNA from *Lactobacillus acidophilus cells* and Kombucha samples was extracted by using the QIAamp^®^cador^®^ Pathogen Mini Kit (Qiagen, Hilden, Germany) and the presence of DNA was verified by electrophoresis with ethidium bromide. The DNA quality was checked using a spectrophotometer (Biophotometer plus, Eppendorf, Hamburg, Germany). The DNA samples were treated with Maxima SYBR Green qPCR Master Mix (Thermo Fisher Scientific, Hampton, NH, USA), in the presence of LAB specific primers LacF (5-AGCAGTAGGGAATCTTCCA-3) and LacR (5-ATTYCACCGCTACACATG-3) recommended by Ritchie et al., 2010 [55]. The reaction was performed in a Real-time PCR System (7900 Fast Real-time PCR System, Applied Biosystems, Thermo Fisher Scientific, Foster City, CA, USA) following the program: UNG (Uracil-N-Glycozylase) treatment 2 min at 50 °C; initial denaturation 10 min at 95 °C; 40 cycles of denaturation 15 s at 95 °C followed by an extension of 60 s at 60 °C. Each sample was amplified in duplicate in every experiment. To generate the standard curve, a 10-fold dilution series of DNA from *Lactobacillus acidophilus* ATCC 4356 was subjected to qPCR under the same conditions as described above. A threshold cycle Ct, for the beginning of exponential phase/detection limit was established at 30. All reagents used were molecular biology grade reagents.

#### 2.6.2. On-plate LAB Counting

The LAB suspensions were counted in Petri dishes with 1% agar MRS (Man, Rogosa, Sharpe, Oxoid), after decimal dilutions, by the incorporation techniques which assure the microaerophilic conditions. The development of the fungi in the medium was inhibited by the addition of cycloheximide (0.5 mg/mL in 96% ethanol). The bacteria were cultivated at 37 °C for 2–3 days, until the colonies could be counted. Reagent suitable for bacteria cultivation was used.

### 2.7. Analysis of Organic Acids

The Kombucha liquid phase was centrifuged at 5000 rpm, for 10 min. and the resultant supernatant was stored at −18 °C. Prior to analysis, the samples were diluted (1:10) with ultrapure water and the diluted samples were passed through 0.2 µm hydrophilic PTFE Millex syringe filter (Merck Millipore, Darmstad, Germany). Two different types of organic acids were analyzed by HPLC: the (poly)hydroxy-acids (lactic, gluconic, citric) and the short chains fatty acids (acetic, propionic and butyric).

#### 2.7.1. Analysis of Hydroxy-Acids

A volume of 10 μL of the filtrate was injected into the HPLC system and the chromatographic analysis was carried out using a Waters Alliance system (Waters Corporation, Milford MA, USA), with a 2695 separation module and a 2487 UV detector (Waters, Milford). The system included a Supelcogel H column (250 mm × 4.6 mm) fitted with Supelcogel H Guard Column (50 mm × 4.6 mm) for the analytic separation, using 0.1% H_3_PO_4_ as mobile phase and0.17 mL/min flow rate. The UV detection was performed at 210 nm. Data were collected and analyzed with the Empower 2.3 system (Waters, Milford). The organic hydroxy-acids were identified according to their retention times: 10.7 min for citric acid, 11.2 min for gluconic acid, 15.1 min for lactic acid. The compounds peak area was used for quantification, based on a calibration curve obtained by injecting different volumes of a standard solution containing 30 ng/µL lactic acid, 25 ng/µL citric acid and 77 ng/µL gluconic acid. LOD/LOQ were 1.2/3.6 ng/µL for lactic acid, 1.1/2.2 ng/µL for citric acid and 1.9/5.7 ng/µL for gluconic acid. All used reagents were HPLC grade reagents, purchased from EMD Millipore, Merck Group.

#### 2.7.2. Analysis of Short Chains Fatty Acids

For this determination an Agilent HPLC 1200 system (Agilent, Palo Alto, CA, USA), comprising a DAD detector, quaternary pump, and a thermostatic auto sampler, was used. A Luna 2 Phenomenex (size: Φ 4.6 × 150 mm) column served as a stationary phase at 25 °C. The mobile phase consisted of: (A) 10mM NaH_2_PO_4_·H_2_O (pH 2.50 ± 0.02) and (B) acetonitrile. A volume of 10 µL was injected into the system and the elution was performed using an isocratic method with 90% (A) and a variable flow: 0.6 mL/min until 1.5 min, 0.4 mL/min at 2 min, 0.25 mL/min from 3 to 15 min, 0.3 mL/min at 16 min, 0.6 mL/min at 17 min, 1 mL/min from 18 to 23 min, 0.6 mL/min until 25 min, for a better separation. The absorbance was monitored at 220 nm. The separation of organic acids was achieved in 25 min and the identification was obtained by comparing their retention times with those of standards using a Chemstation (Agilent, Palo Alto, CA, USA) software. All used reagents were HPLC grade reagents, purchased from EMD Millipore, Merck Group.

### 2.8. Ultrastructural and Morphological Analysis

For ultrastructural analysis, the fermented pollen samples were investigated by transmission electron microscopy (TEM), using a Philips EM 208S electron microscope equipped with Veleta video camera and imaging software iTEM Olympus Soft Imaging System. The morphological analysis of the samples was performed by scanning electron microscopy (SEM), using a SEM-HITACHI SU-1510 microscope. The samples were centrifuged at 10,000 rpm and the resultant sediments were subjected to a plant-specific fixation process with 3% glutaraldehyde, 1.5% paraformaldehyde, 1 M Na_3_PO_4_, for 2 h, to create cross-links between the constituent molecules of the processed material and to avoid the destruction of the ultrastructural architecture. The fixation step was followed by rinsing with 0,5 M Na_3_PO_4_, 3 times at 4 °C. For the post-fixation step the sample was transferred in a solution of 1% OsO_4_ and 0.5 M Na_3_PO_4_, for 1 h in the dark. After the post-fixation step, the samples were washed in water at 4 °C (3 times for 10 min) and dehydrated by successive washings with ethanol, 10 min each: 12.5%, 25%, 35%, 50%, 70%, 80%, 90%, 95% and 100%. To replace the old solution and ethanol completely with an acetone-resin mixture, the dehydrated samples were incubated successively with acetone-resin (1:1) for 1 h, acetone-resin (1:2) for ½ h, resin 100% for 1 h, and again fresh resin 100% over night. Next, the sample was placed in fresh resin (100%), for 60 h at 50 °C, to perform the polymerization step. The samples were cut with an ultramicrotome Leica UC6 (Leica Biosystem, Wetzlar, Germany), with a diamond knife and the resultant sections were placed on copper grids of 200 mesh, covered with a formvar pellicle. The samples were stained for 7 min with 5% uranyl acetate in absolute methanol, to increase the contrast. Then the samples were washed in distilled water and re-stained for another 7 min, with a solution of lead nitrate 4.4%, 0.2 M trisodium citrate monohydrate in distilled water (with 1% NaOH to clarify the solution). All reagents were of electron microscopy grade, supplied by Sigma-Aldrich (Sigma, Merck Millipore, Darmstadt, Germany).

### 2.9. Biocompatibility and Antitumoral Activity

The spray dried product resultant from the process scale-up in 100 L fermentation vessels was evaluated in vitro, for biocompatibility, on normal mouse fibroblast cell line, NCTC clone L929, and for antitumoral activity, on two transformed human cell lines, laryngeal epidermoid carcinoma cells (Hep-2) and human colon adenocarcinoma cells (Caco-2). The cells were maintained in minimum essential medium (MEM) supplemented with 10% fetal bovine serum and 1% antibiotics (penicillin, streptomycin and neomycin) at 37 °C, in a humidified atmosphere with 5% CO_2_. The spray dried Kombucha fermented pollen was solubilized in MEM at a concentration of 50 mg/mL and this stock suspension was sterilized by passage through a 0.2 µm hydrophilic PTFE Millex syringe filter (Merck Millipore). The cells were treated with different concentrations of the suspension (5, 6, 8, 10, 15, 20, 25 and 30 mg/mL) and the treated cells were incubated for 24 and 72 h at 37 °C, in a humidified atmosphere with 5% CO_2_. All reagents used were suitable for cell culture and were purchased from Sigma–Merck Group.

#### 2.9.1. Cell Viability

The cell viability was assessed by the MTT (3-(4,5-dimethylthiazol-2-yl)-2,5-diphenyltetrazolium bromide) assay. This assay, based on the reduction of a yellow tetrazolium salt into purple formazan dye by active mitochondria [56], was adapted to our laboratory conditions [57]. Briefly, the cells were seeded in 96-well culture plates, at a density of 5 × 10^4^ cells/mL (for L929 and Hep-2) and 1 × 10^5^ cells/mL (for Caco-2) and incubated at 37 °C, to allow cell adhesion. After 24 h, the culture medium was discarded and replaced with fresh medium, containing different concentrations of spray-dried Kombucha fermented pollen. After 24 and 72 h of incubation, the cells were rinsed with phosphate buffered saline solution (PBS), pH 7.4, and incubated with 100 μL of MTT working solution (0.25 mg/mL) for 3 h at 37 °C. At the end of incubation period the medium was removed, and the formazan crystals were dissolved in 100 μL isopropanol. The optical density of the solution was measured after 15 min of incubation at room temperature, with gently stirring, using a microplate reader (Sunrise, Tecan, Männedorf). The concentration of converted MTT dye directly correlates with the number of metabolically active cells. The cell viability was expressed as a percentage of the control cells (untreated cells) considered 100% viable. All samples were tested in triplicate. All reagents used were suitable for cell culture and were purchased from Sigma–Merck Group.

#### 2.9.2. Cell Morphology

The culture plates of normal L929 cells and of the transformed Hep-2 and Caco-2 cells were examined after 72 h of incubation in the presence of the pollen suspension. Images of the cells were acquired using a Zeiss Axio Observer microscope, with Axio Vision 4.6 software (Carl Zeiss, Oberkochen, Germany).

### 2.10. Statistical Analysis

Each assay was performed in triplicate. The Student t-test from the SPPS 21 Software package (IBM, Armonk, NY, USA) was used to analyze the relevance of the quantitative data, with the significance level set at *p* < 0.05.

## 3. Results

The major components of the multi-floral bee collected pollen were from the families: Brassicaceae (*Brassica* sp.) Asteraceae (*Carduus* sp., *Helianthus annus*, *Taraxacum officinale*), Rosaceae (*Prunus* sp., *Crataegus* sp.), Malvaceae (*Tilia* sp.) and Poaceae (*Zea mays*). Such composition is similar to that reported for other batches of multi-floral, bee collected pollen, from the same region of Romania [58]. 

### 3.1. Release of Pollen Biosilica, Polyphenols and Flavonoids During Fermentation with Kombucha

**Figure 1:**
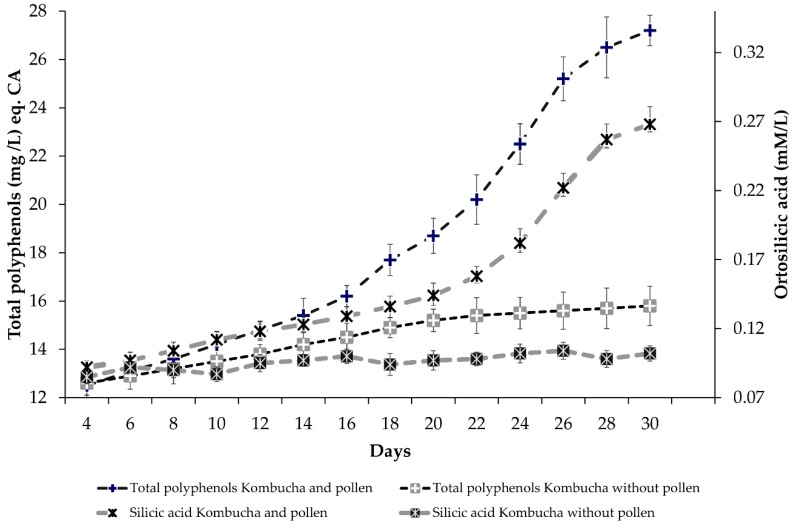
Evolution of soluble silicon and total polyphenols in Kombucha liquid phase, with and without addition of bee collected pollen.

The total polyphenol content of the bee collected pollen batches used for our experiments was 12.73 ± 3.38 mg/g dry weight (d.w.). Such content is in line with those reported already for Romanian pollen [59]. We determined a total silicon content of 4.47 ± 0.78 mg/g d.w., on the same level as the reported values for pollen from the same geographic region—Eastern Europe/Asia Minor [60,61]. The biosilica from pollen wall was solubilized during Kombucha fermentation. The solubilization was more significant after 18 days of fermentation (Figure 1) and it is probably a result of the accumulation of organic acids [62].

The release of polyphenols into Kombucha liquid phase during the fermentation of pollen followed the same pattern as the solubilization of biosilica, with a more significant and accelerated release after 15–18 days of fermentation (Figure 1). It was already reported that the content of polyphenols is increased during Kombucha fermentation [63]. The addition of another vegetal source of polyphenols, i.e., wheatgrass juice, significantly increased the polyphenol content of the Kombucha [64].

**Figure 2:**
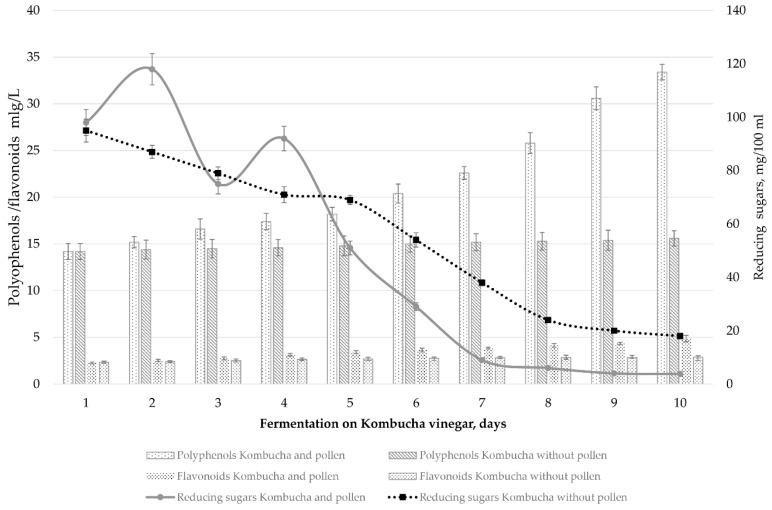
Evolution of polyphenols, flavonoids and reducing sugars of Kombucha vinegar wherein bee collected pollen was introduced.

The phytonutrients from pollen are released mainly after 18–20 days of fermentation in the Kombucha liquid phase. The addition of pollen into Kombucha vinegar after 20 days of fermentation determined a significant increase in the content of total polyphenols and flavonoids in the liquid phase (Figure 2). At the same time, the content of reducing sugars decreased, after a small increase during the first days (Figure 2), showing that the microorganisms from the Kombucha consortium are still active during this late fermentation stage. The small increase in sugar reducing content during the first days after pollen addition suggests a release of such compounds from pollen.

The values of the DPPH and TEAC assays for each day of pollen fermentation in Kombucha vinegar are presented in Table 1, and they show an increase in the recorded values, and therefore of the antioxidant activity as the fermentation process unfolds. The antioxidant activity profile had a similar pattern of evolution to that of the total content of polyphenols and flavonoids. The IC50 value, calculated for the DPPH free radical scavenging activity, decreased continuously from the first fermentation day to the 10^th^ day.

### 3.2. Dynamic of Lactic Acid Bacteria Population During Product Fermentation

LAB were previously reported to comprise up to 30% of the bacterial population of Kombucha cultures/SCOBY consortium [32]. In our study we used qPCR to quantify the LAB in different Kombucha samples with or without added pollen. The molecular data were compared with results obtained by on-plate cultivation of LAB in specific media, respectively MRS.

The first applications of qPCR in microbial ecology were reported in 2000, and it is still considered a fast and effective method enabling the quantification of transcript numbers within environmental samples, providing high specificity and sensitivity to targeted sequences present in mixed community background using specific primers [65]. This method has been previously used for detection of lactic acid bacteria dynamics during fermentation, such as *Streptococcus thermophilus* and *Lactococcus lactis* in dairy [66], *L. plantarum* and *L. fermentum* in cocoa bean fermentation [67], *L. curvatus*, *L. brevis*, *L. pontis*, *Pediococcus pentosaceus* in sourdough [68,69]. Under our experimental conditions, the correlation coefficient was *R*^2^ = 0.9948 for the standard curve. The detection limit showed a maximum Ct (threshold cycle, related to the number of amplification cycles needed for the exponential amplification phase to begin) of 30, which was needed for a positive reaction with SYBR-Green I. The amount of LAB specific DNA present in an unknown sample was obtained by interpolating its Ct value against the standard curve, and it was expressed in log10 CFU/mL.

**Figure 3:**
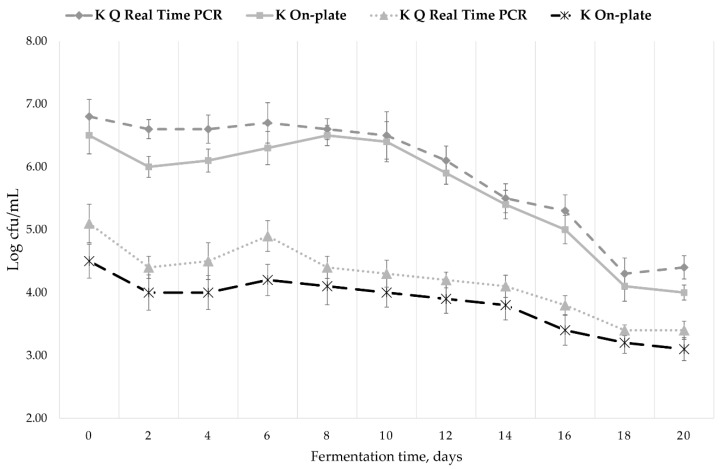
Lactic acid bacteria population during Kombucha without (K) or with pollen (KP) fermentation. Data obtained by q-Real Time PCR versus on-plate quantification.

As seen in Figure 3, immediately after inoculation with SCOBY during Kombucha fermentation, the level of LAB measured by both methods is close to 10^5^ CFU/mL, level which remains constant during the first five fermentation days and goes down to 10^3^–10^4^ CFU/mL by the end of the fermentative process. When adding pollen in the beginning of the fermentation, the initial LAB level raised almost two logarithmic units, up to 10^7^ CFU/mL.

For the initial level of LAB in the pollen, three different samples of multi-flower pollen used on an industrial level were investigated by q-PCR technique. The LAB content varied between 10^5^–10^6^ CFU/mL total cells. Until now, few studies have focused on pollen LAB payload. Our result is in line with the only data already reported, respectively a level of viable cells (on-plate counting) from 10^4^–10^6^ CFU/mL [65]. The most common specie identified by molecular tools in the pollen was reported previously to be *Lactobacillus* species and in particular *Lactobacillus kunkeei* [66]. Therefore, the important bounce of the lactic acid bacteria population upon pollen addition could be caused by the pollen contribution, considering the reports which emphasize such transfer of lactic bacteria of genus *Lactobacillus* sp, *Bifidobacterium* sp. from the bees’ digestive system into the bee pollen, being involved in bee bread fermentation [21,67].

Since we did not register significant differences between the level of LAB measured by qPCR and on-plate technique (colony forming units), respectively, between the total LAB cells and the viable LAB cells, we will only use qPCR in further studies, as it is faster and more reliable.

**Figure 4:**
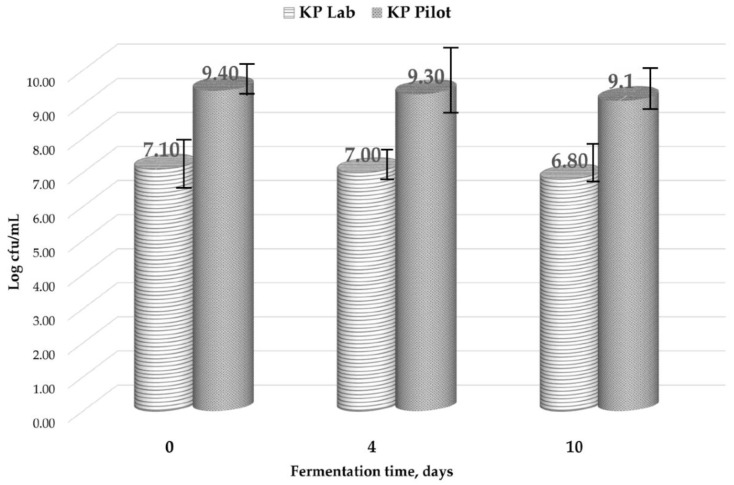
Lactic acid bacteria level during the fermentation process of Kombucha with pollen. Laboratory (Lab) versus Pilot Plant (Pilot) scale.

When adding pollen in Kombucha vinegar (Kombucha previously fermented for 20 days) we did not notice significant differences in LAB population level between the first and the last maceration day. The experiment on the laboratory scale in 2 L working volume induced a lower level (10^7^ CFU/mL) than that resulted from pilot trials of 100 L (10^9^ CFU/mL) (Figure 4). This may be linked to the specific ratio area/volume and interfacial specific area on large scale level, which assure better conditions for LAB development. Up-scale enhances the pollen effect of boosting LAB microbial population. Further investigations should be performed in this respect.

### 3.3. Dynamics of Organic Acids During Product Fermentation

We determined the dynamics of several organic acids, hydroxy acids (citric, gluconic and lactic) and short-chain fatty acids (acetic, propionic and butyric). Pollen addition determined an enhancement in the production of the organic acids (Table 2).

Such enhancement of organic acid production is most probably related to the significant increase of SCOBY biodiversity, with a higher weight/proportion of LAB. Different types of fermentation occur at the same time in Kombucha with pollen. As seen in Table 1, citric acid was not detectable (ND) in any of the samples without pollen and only in the case of Kombucha fermented with pollen (KP) some small values were detected, increasing from 0.02 g/L in the beginning of the fermentation to 0.055 g/L by the end of the fermentation. The presence of citric acid in Kombucha was previously reported by Jayabalan et al. [31], who found a content of 0.03 g/L in Kombucha prepared with green tea, on the 3rd day of fermentation and 0.11 g/L in the product obtained from black tea. Regarding lactic acid, higher levels were obtained in the samples fermented with pollen, almost double in the final product (0.37 g/L without pollen and 0.7 g/L with pollen), which could be correlated with the higher levels [68] of lactic acid bacteria population brought by the pollen addition. Malbasa et al. (2008) [31] reported a maximum of 0.54 g/L *L*-lactic acid in Kombucha made of black tea, which is in the same range with our results.

In the product fermented without pollen, the gluconic acid content increased by the end of the processes, reaching 1.5 g/L, while when adding pollen, a small increase in the first 9 days was noticed, followed by its decrease. Gluconic acid is produced mainly by acetic bacteria and the high level of lactic acid bacteria may inhibit the growth of acetic bacteria. Chen and Liu (2000) [69] reported a higher content in Kombucha made with black tea, 39 g/L, but after a much longer fermentation period.

Acetic acid is the main organic acid in all samples and increases during the fermentation, due to the activity of acetic bacteria. In the laboratory samples, fermented with and without pollen, the final content of acetic acid was 3.51 and 4.61 g/L, respectively, which is in the range with the data already reported [63], respectively 4.69 g/L after 18 days of fermentation. However, the samples obtained under pilot plant conditions contained a substantial higher level of acetic acid than laboratory samples, reaching levels of 17–19 g/L acetic acid, most probably due to the same surface-volume difference between laboratory and larger scale level.

To the best of our knowledge, the presence of propionic and butyric acids has not been reported yet in Kombucha beverages. However, the presence of these SCFA is plausible and it was probably not reported because it was not extensively studied. Such SCFA are usually produced by the probiotic microorganisms, including lactic acid bacteria, and are an important part of postbiotics [36,37]. The higher content of LAB within SCOBY developed by tea with pollen could explain the higher production of such SCFA in Kombucha with pollen.

### 3.4. Morphological and Structural Analysis of Fermented Pollen

**Figure 5:**
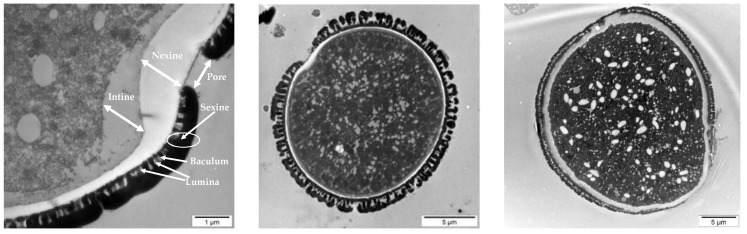
Control sample of unfermented pollen. The arrows show the external wall exine, consisting of sexine and nexine, and the inner wall intine. Additional ultrastructural characteristics of pollen, lumina and baculum are spotlighted. Middle and right pictures illustrate representative aspects of the analyzed pollen grains.

**Figure 6:**
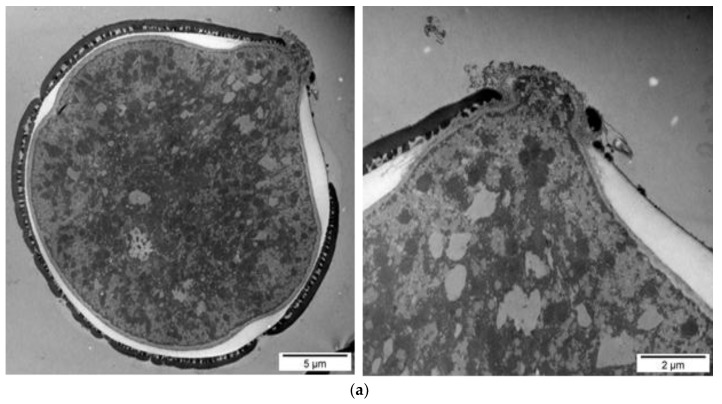

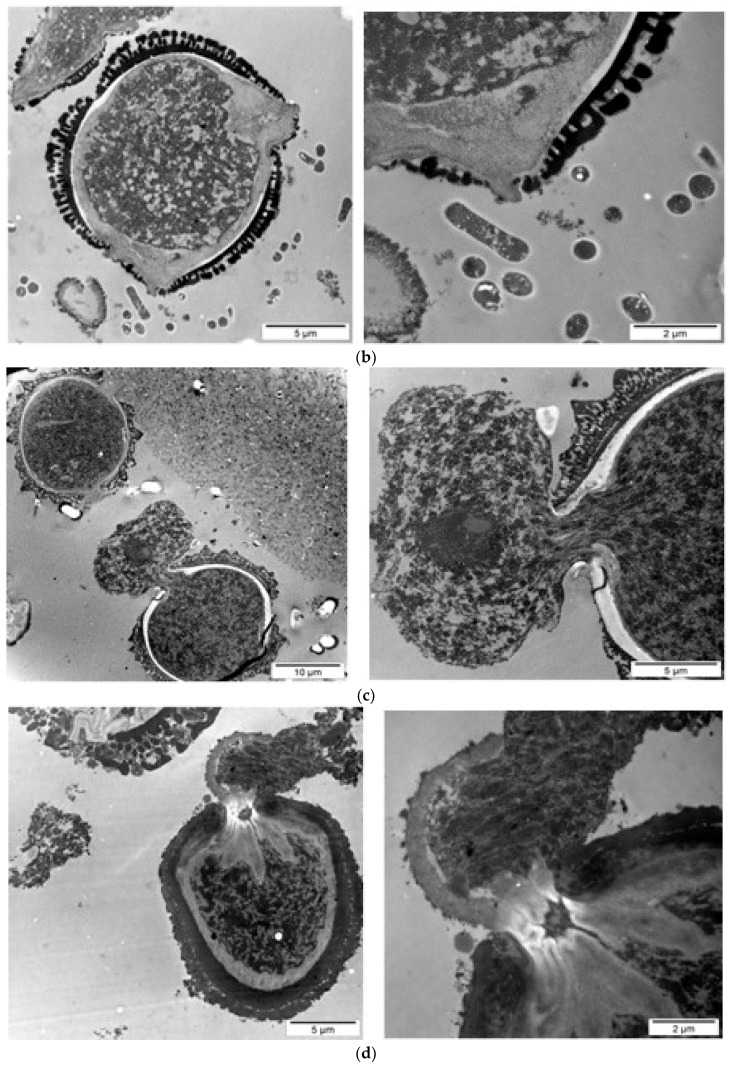
Fermented pollen in kombucha vinegar: (**a**) 3 days, (**b**) 5 days, (**c**) 7 days, (**d**) 9 days.

The ultrastructure analysis with transmission electron microscopy (TEM) highlighted differences between the control (dry) pollen (Figure 5) and pollen undergoing a fermentation process in Kombucha vinegar, for samples at 3rd, 5th, 7th and 9th fermentation day (Figure 6). A very important feature of the pollen grain is the resistant outer coat named exine, which has a role in protecting the reproductive cells and is important for the attachment to insect pollinators and adhesion to the stigmatic surfaces. The exine is composed of sexine—the ornamental external part, and the nexine, the basal layer. Beneath the exine, a second major wall layer, the intine, surrounds the pollen grain protoplasm. The exine is composed of sporopollenin, a highly resistant biopolymer containing fatty acids, phenylpropanoids, phenolics and carotenoids, and the intine is largely composed of pectin and cellulose.

There is a progressive dynamic of destruction of the pollen complex wall structure, starting both from exterior and interior of the pollen grain. TEM images show that there are visible modifications of the structural units of the pollen shells (external and internal pollen coating—exine and intine). The pollen grains undergo wall destruction and release of the cellular content in the Kombucha vinegar.

During the first days of fermentation the swelling of the pollen granule, the breaking of the exine and the opening of the pores were observed (Figure 6a,b). Planktonic bacteria are present near the pollen grain (Figure 6b). Most probably, the observed bacilli and cocci are lactic acid bacteria, e.g., *Lactobacillus* sp. or *Pediococcus* sp.—our group recently reported the presence of *Pediococcus pentosaceus* in our local SCOBY/Kombucha consortium. Our hypothesis is that these planktonic bacteria are involved in the breakage of the pollen complex wall structure.

The exine starts to erode and to break, further exposing the pores and the intine. The swelling, created also due to the difference in osmotic pressure, generates an internal pressure, which is pushing on pores. As we approach the 10th day of fermentation, we notice the extensive breakage of the intine. The intine destruction results in significant release of the internal content (Figure 6c,d). Our observations on ultrastructural changes support the biochemical results which show an increase in the content of bioactive compounds (flavonoids, polyphenols, antioxidant activity) in Kombucha liquid phase wherein pollen is included, as compared with the Kombucha without pollen. Most probably, the additional bioactive ingredients from Kombucha fermented with pollen are released from the pollen grains that undergo destruction of the complex wall structure.

**Figure 7:**
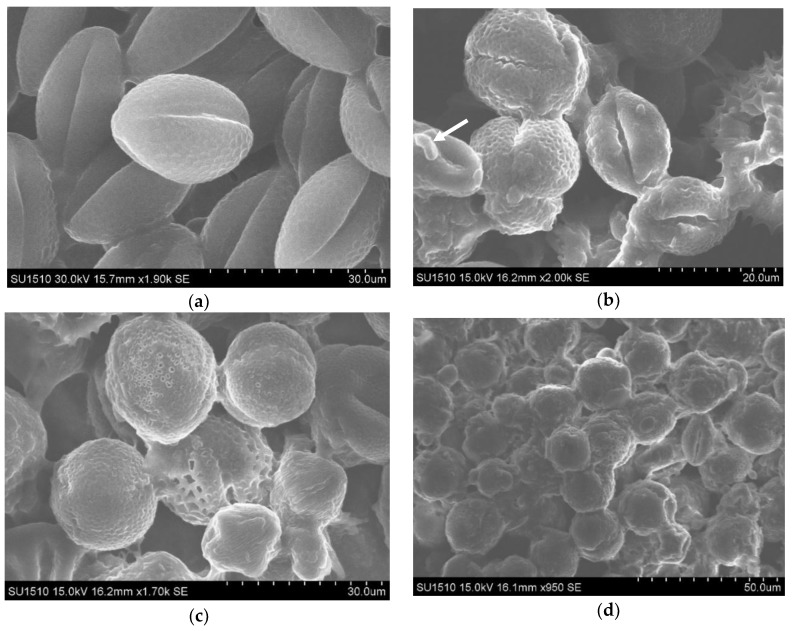

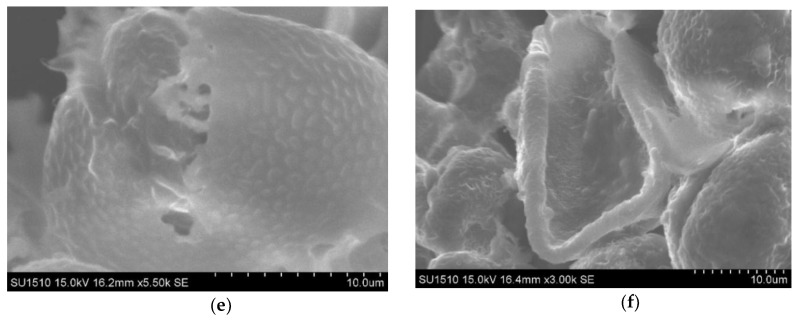
SEM evaluation of the ultrastructure of mono-floral, apple pollen, which has a more uniform shape. (**a**) unfermented pollen; (**b**) 1-day fermented pollen, with attached bacteria (arrow); (**c**,**d**) 7 days fermented pollen; (**e**) detail: pollen granule cracked after 10 days of the fermentation process; (**f**) detail: pollen granule drained because of the10 day of fermentation process.

SEM images highlight the adhesion of the bacteria from SCOBY consortium microorganisms to the surface of the pollen granules (Figure 7b, arrows). Such a finding supports our hypothesis regarding the involvement of the planktonic microorganisms from Kombucha/SCOBY consortium (the microorganisms which are not included into the cellulose biofilm) in the pollen degradation. Most likely, different microorganisms from Kombucha/SCOBY consortium develop on the surface of pollen grains and promote the formation of small pores into exine, through local oxidative degradation. The SEM analysis also illustrates the morphological changes of pollen granules after Kombucha fermentation, such as swelling and tearing.

Both the morphological and ultrastructural analyzes present evidences for an increased bioavailability of the pollen grain content after fermentation with SCOBY/Kombucha consortia. Such a fermentation process determines the morphological and ultrastructural alterations of the pollen outer and inner membrane and leads to the release of the pollen granule content.

### 3.5. Cytotoxic and Antitumoral Effects of Spray-Dried Product

**Figure 8:**
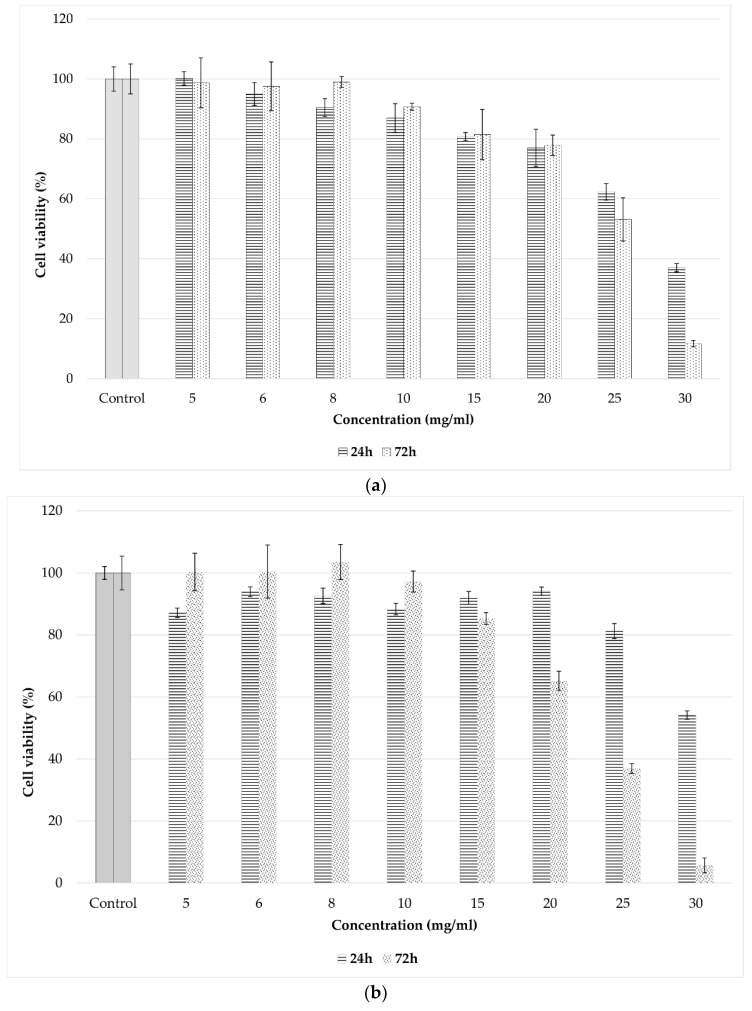

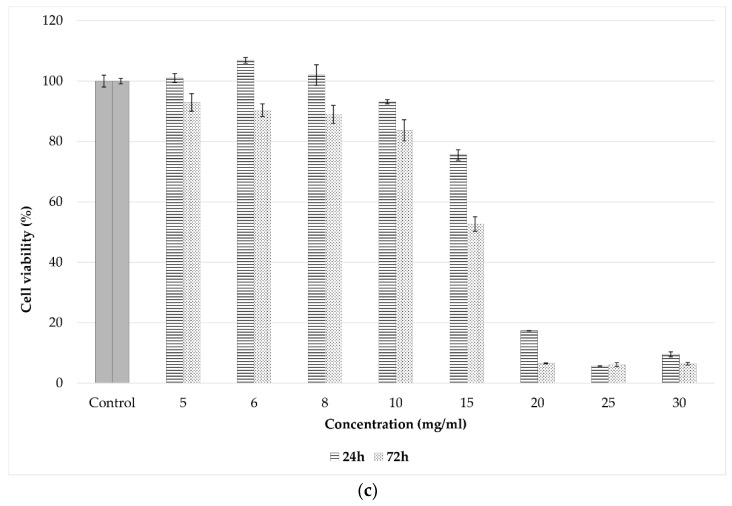
Effect of treatment with the spray-dried, large-scale obtained product on cell viability of (**a**) NCTC clone L929, (**b**) Hep-2 cells and (**c**) Caco-2 cells, after 24 and 72 h, evaluated by the MTT assay. The experiment was carried out in triplicate. Data are presented as mean ± SD.

We used spray-dried pollen processed on pilot plant level, 100 L working volume, to determine the cytotoxic and antitumoral effects. The in vitro biocompatibility of the spray-dried sample was assessed using the MTT assay, which evaluates the activity of mitochondrial dehydrogenases. The results obtained after 24 h and 72 h of cell incubation in the presence of different concentrations of the sample indicated that the spray-dried product (fermented pollen with Kombucha consortium soluble products, metabolites and cell components) presented a good biocompatibility within the concentration range of 5–20 mg/mL. At these concentrations, the cell viability was above 80%, which is considered non-cytotoxic (Figure 8a). At concentration of 30 mg/mL, the cell viability decreased to 37% and 11.71% after 24 h and 72 h of treatment, respectively. The extracts/products added into the cell culture media at concentrations higher than 2%, respectively 20 mg/mL, modify the characteristics of the cells.

Regarding the antitumoral activity, after 24 h of treatment, the viability of Hep-2 cells was maintained over 80% at concentrations ranging between 5–25 mg/mL but dropped significantly below 65% after 72 h starting with the concentration of 20 mg/mL (Figure 8b). Better results were obtained on Caco-2 intestinal tumor cells. Thus, the values of cell viability slightly decreased below 80% (75.56%) after 24 h of treatment at the concentration of 15 mg/mL, whereas at higher concentrations, significantly lower values were obtained (below 20%) (Figure 8c). Similar results were found after 72 h, although the values of the cell viability were even lower at all tested concentrations compared to those obtained after 24 h. Thus, the cell viability dropped to 52.65% at the concentration of 15 mg/mL and below 10% at higher concentrations (20–30 mg/mL).

In conclusion, the tested sample showed no cytotoxic effect within the concentration range of 5–20 mg/mL and exhibited a moderate antitumoral activity starting with the concentration of 20 mg/mL for Hep-2 cells and 15 mg/mL for Caco-2 intestinal tumor cells.

#### Cell Morphology

Optic microscopy allowed for observations of cell morphology changes induced in the cell membrane, cytoplasm and nuclei after treatment with different concentrations of atomized sample. Light microscopy images were taken for all three types of cell lines (NCTC clone L929, Hep-2 and Caco-2) incubated in the presence of the tested sample for 72 h (Figures S1–S3). The micrographs of the NCTC clone L929 control culture showed that the cells maintained their normal fibroblastic phenotype with euchromatic nuclei and clear cytoplasm (Figure S1a). The morphology of L929 cells treated with the spray-dried product was similar to that of the untreated control up to the concentration of 20 mg/mL and the cell density reached a complete monolayer similar to the control cells (Figure S1b). Evident morphological changes in the cell morphology, such as round cells and giant cells with multiple nuclei and granular cytoplasm, were observed at higher concentrations (25–30 mg/mL), when the cell viability dropped below 55% (Figure S1c,d).

The light microscope images of Hep-2 cells cultivated in the presence of the spray-dried product revealed a normal morphology (epithelial-like cells) like that of the untreated cells up to the concentration of 15 mg/mL (Figure S2a,b). Moreover, the density of treated cells was also like that of the control cells, reaching an almost complete monolayer (90–95% surface covered by cells). Morphological changes in the cell membrane, cytoplasm and nuclei were observed starting with the concentration of 20 mg/mL, when cell viability decreased significantly, down to 5.66% at the concentration of 30 mg/mL (Figure S2c,d).

Finally, Caco-2 cells treated with different concentrations of the product showed a normal epithelial-like phenotype (cuboidal shape, similar to intestinal enterocytes) just as the untreated cells, within the concentration range of 5–10 mg/mL, when the cell density reached an almost complete monolayer (85–90% surface covered by cells) (Figure S3a,b). Significant morphological modifications were observed at higher concentrations, with an increased percentage of rounded cells and low cell density (Figure S3c,d).

In conclusion, the quantitative results obtained by the MTT assay correlated well with cell morphology observations based on optical microscopy and both highlighted the antitumoral effect of the tested sample.

## 4. Discussion

The results demonstrate that the process which we proposed, fermentation of bee collected pollen with Kombucha, increases the bioavailability of bioactive compounds from pollen. The fermentation of pollen with Kombucha releases important amounts of soluble silicon from biosilica embedded into the wall. It is known that plant biosilica presents two pools, a concentrated one, in the form of SiO_2_*x*nH_2_O aggregates and a dispersed one, poly-condensed silica complexed within the cell wall [70]. Concentrated forms of biosilica aggregates have not been described in pollen. Thus, most probably, the soluble silicon species which are released from pollen into Kombucha liquid phase result from the dissolution of pollen wall matrix in the liquid phase. Weakening of the pollen wall due to silicon solubilization increased the amount of released nutrients into Kombucha liquid phase. We found an increase in the polyphenols content during the pollen fermentation with Kombucha. Pollen proteins/amino acids are also most probably released after pollen grain breakage. Both polyphenols and amino acids were reported to solubilize biosilica through a surface complexation reaction [71]. This suggests an interconnected, positive feed-back process, by which compounds released from pollen could accelerate further the dissolution of wall matrix. Such processes could explain the exponential phase release of pollen bioactive compounds, which we found both for pollen fermented in Kombucha from the very beginning and for pollen fermented/macerated in Kombucha vinegar.

Pollen addition to Kombucha determined a better development of LAB. In Kombucha fermented on industrial scale, the metabarcoding technique from a previous study revealed values from 6 to 8 units log CFU/mL, in the case of fermentation of green tea, from the 1st to the 8th day of fermentation [72]. In our experimental large-scale fermentation of Kombucha with pollen, for 100 L working volume, we determined more than 9 units log CFU/mL, even on the 10th day of fermentation. The larger weight of the LAN population into SCOBY consortium from Kombucha with added pollen is correlated with higher formation of lactic acid.

Large-scale fermentation of pollen with Kombucha proved to be more effective than laboratory, small scale batches. The main reason is probably related to the interfacial transfer of oxygen. During Kombucha fermentation the SCOBY consortium accomplishes different types of fermentation in the same time, an aerobic one (acetic acid fermentation, oxygen being needed to transform ethanol to acetic acid) and anaerobic fermentations—alcoholic and lactic [73]. Oxygen transfer should be enough for acetic acid fermentation, but not in excess to inhibit alcoholic and/or lactic acid fermentation. The oxygen transfer rate in the static culture of Kombucha is determined by the interfacial specific area [74], i.e., the ratio between the surface exposed to air/oxygen and the total working volume. The higher this ratio, the faster is the rate of oxygen transfer and the production of acetic acids. However, on a too large area, the oxygen level in fermentation broths/liquid phase could limit significantly the anaerobic fermentation. Thus, there are some limits for interfacial specific area, between 0.0232 and 0.0681 cm^−1^, for a proper Kombucha fermentation process [75]. The vessel which we used for large-scale pollen fermentation with Kombucha has a specific interfacial ratio of 0.0573 cm^−1^, which assures better oxygen transfer than the cylindrical bottles used under laboratory conditions, with a specific interfacial ratio of 0.0308 cm^−1^.

The addition of pollen seems to increase the proportion of lactic acid producers. The better development of LAB on large scale fermentation with Kombucha could be explained by the larger microbiota diversity associated with the diverse bee collected batches used on large scale. It has been already demonstrated that introduced LAB are well tolerated by SCOBY consortium; For the hybrid Kombucha culture, sweetened tea—sweetened cabbage brine, the microbial consortium maintained the large amount of LAB introduced by the cabbage brine [76]. Most probably, bee collected pollen brings to the SCOBY community fructophilic lactic acid bacteria, specific to honey bee and honey bee products [77]. Fructophilic lactic acid bacteria are fitted to establish and develop into SCOBY consortium/Kombucha habitat: are tolerant to oxygen and high level of sugars [75], metabolize fructose [77] and use polyphenols as electron acceptors [78]. All these features support them to proliferate into sweetened tea, with high content of soluble sugars (including fructose, produced by yeast invertase from sucrose) and polyphenols. Additional polyphenols released from pollen should further support fructophilic LAB. Further studies will help to elucidate these observations.

Larger microbial diversity of Kombucha with added pollen supports also the formation of short-chain fatty acids—SCFA. SCFA are representative for postbiotics, bioactive metabolites produced by probiotics [36]. Postbiotics are among the main active ingredients of paraprobiotics, products which contain inactivated probiotics/beneficial microbial strains. Typical examples of paraprobiotics are the milk-based product fermented with *Bacteroides xylanisolvens* DSM23964. Such products result from cultivation of *B. xylanisolvens* on (skimmed) milk, followed by a thermal inactivation (including by spray-drying) of the resultant fermentation product. Spray-drying in mild conditions does not affect the bioactive compounds produced by *B. xylanisolvens* and reduces the concerns regarding potential hazards of active growing microbial strains for consumers with (auto)immune disorders and inflammatory conditions. We used the same approach to limit the concerns regarding the safety of our pollen fermented with Kombucha. The cell culture tests proved a good biocompatibility. Even after inclusion of 20 mg/mL in the culture media (i.e., 2%) the spray-dried pollen fermented with the Kombucha soup is still biocompatible. Usually, the biocompatibility of herbal extracts on cell culture of NCTC clone L929 is investigated at concentrations between 0,1 and 0,5%, respectively from 1 to 5 mg/mL, and the products with limited influence at 0.5% concentration on growing medium are considered safe [79]. In the case of transformed cells of intestinal origins, we noted an inhibitory effect starting from 10–15 mg/mL. This antitumoral slight effect might be related to the presence of postbiotics into the tested products, as such compounds were demonstrated to have antitumoral effect against intestinal transformed cells [36].

## 5. Conclusions

The fermentation with a Kombucha consortium enhances pollen phytonutrients bioavailability. However, the pollen fermentation with Kombucha leads not only to enhanced bioavailability of pollen phytonutrients. Pollen has already been demonstrated to be a good fermentation activator for mead and white wine. Our data presented here show that pollen is also a good activator of Kombucha/SCOBY fermentation. The addition of the bee collected pollen also improves Kombucha fermentation and the formation of Kombucha health-related compounds. At the end of the pollen fermentation with Kombucha consortium, a product with enhanced health benefits is formed, with complementary bioactive ingredients.

## Figures and Tables

**Table 1:** Time-dependent antioxidant capacity of Kombucha vinegar with added pollen.

Fermentation Time (days)	TEAC (µg Trolox/mL)	DPPH (Inhibition Grade%/mL)	IC_50_ (mg/mL)
1	8.83 ± 0.17	1.35 ± 0.1	15.16
2	3.54 ± 0.63	1.32 ± 0.13	13.76
3	3.9 ± 0.18	1.62 ± 0.04	14
4	4.21 ± 0.21	1.64 ± 0.03	12.6
5	5.61 ± 0.22	2.16 ± 0.06	14.96
6	7.8 ± 0.26	2.85 ± 0.07	13.76
7	7.23 ± 0.31	3.79 ± 0.07	11.97
8	16.24 ± 0.34	4.87 ± 0.04	11.95
9	20.94 ± 0.67	4.86 ± 0.11	11.68
10	22.95 ± 0.77	4.91 ± 0.11	10.56

**Table 2:** Organic acids content during Kombucha fermentation with or w/o pollen addition.

Sample	Day	Hydroxy-Acids			Short-Chain Fatty Acids		
		Citric Acid, g/L	Gluconic Acid, g/L	Lactic Acid, g/L	Acetic Acid, g/L	Propionic Acid, g/L	butyric Acid, g/L
K Lab	0	ND	0.545 ± 0.006	0.38 ± 0.01	0.375 ± 0.005	ND	ND
	5	ND	0.56 ± 0.006	0.36 ± 0.006	0.605 ± 0.004	ND	0.14 ± 0.011
	9	ND	0.555 ± 0.014	0.36 ± 0.006	1.32 ± 0.03	0.12 ± 0.033	0.18 ± 0.014
	13	ND	0.94 ± 0.03	0.37 ± 0.006	2.32 ± 0.015	0.16 ± 0.024	0.28 ± 0.017
	17	ND	1.59 ± 0.043	0.375 ± 0.003	4.66 ± 0.025	0.24 ± 0.016	0.30 ± 0.021
KP Lab	0	0.02 ± 0.005	2.795 ± 0.015	0.46 ± 0.015	0.415 ± 0.005	0.095 ± 0.012	0.12 ± 0.038
	5	0.02 ± 0.005	3.155 ± 0.016	0.44 ± 0.01	0.515 ± 0.007	0.205 ± 0.034	0.44 ± 0.032
	9	0.045 ± 0.005	3.59 ± 0.090	0.57 ± 0.02	2.275 ± 0.013	0.31 ± 0.023	0.74 ± 0.028
	13	0.04 ± 0.003	2.545 ± 0.005	0.72 ± 0.01	2.25 ± 0.04	0.42 ± 0.014	1.06 ± 0.063
	17	0.055 ± 0.005	2.26 ± 0.02	0.77 ± 0.01	3.51 ± 0.11	0.56 ± 0.041	1.78 ± 0.054
K Pilot	18	ND	2.645 ± 0.14	1.75 ± 0.15	17.64 ± 0.21	0.27 ± 0.032	0.84 ± 0.037
KP Pilot	18	0.09 ± 0.001	3.975 ± 0.32	10.265 ± 0.42	19.56 ± 0.18	0.66 ± 0.037	1.92 ± 0.033

*Legend:* K—fermented Kombucha; KP—Kombucha fermented with pollen; Pilot—large-scale.

## Supplementary Materials

The following are available online at http://www.mdpi.com/2072-6643/10/10/1365/s1, Figure S1: Light micrographs of NCTC clone L929 cells untreated (a) and treated with different concentrations of atomized product: 20 mg/mL (b), 25 mg/mL (c) and 30 mg/mL (d); Figure S2: Light micrographs of Hep-2 cells untreated (a) and treated with different concentrations of atomized product: 15 mg/mL (b), 20 mg/mL (c) and 30 mg/mL (d); Figure S3: Light micrographs of Caco-2 cells untreated (a) and treated with different concentrations of atomized product: 10 mg/mL (b), 15 mg/mL (c) and 20 mg/mL (d).
