# Supplementary material for: Bee Collected Pollen with Enhanced Health Benefits, Produced by Fermentation with a Kombucha Consortium
Source: Nutrients. 2018 Sep 23;10(10):1365. doi: 10.3390/nu10101365 (PMC6213263; doi:10.3390/nu10101365)
Supplement: Supplementary file 1 [file nutrients-10-01365-s001.pdf]

Article

Bee collected pollen with enhanced health benefits, produced by fermentation with a Kombucha consortium

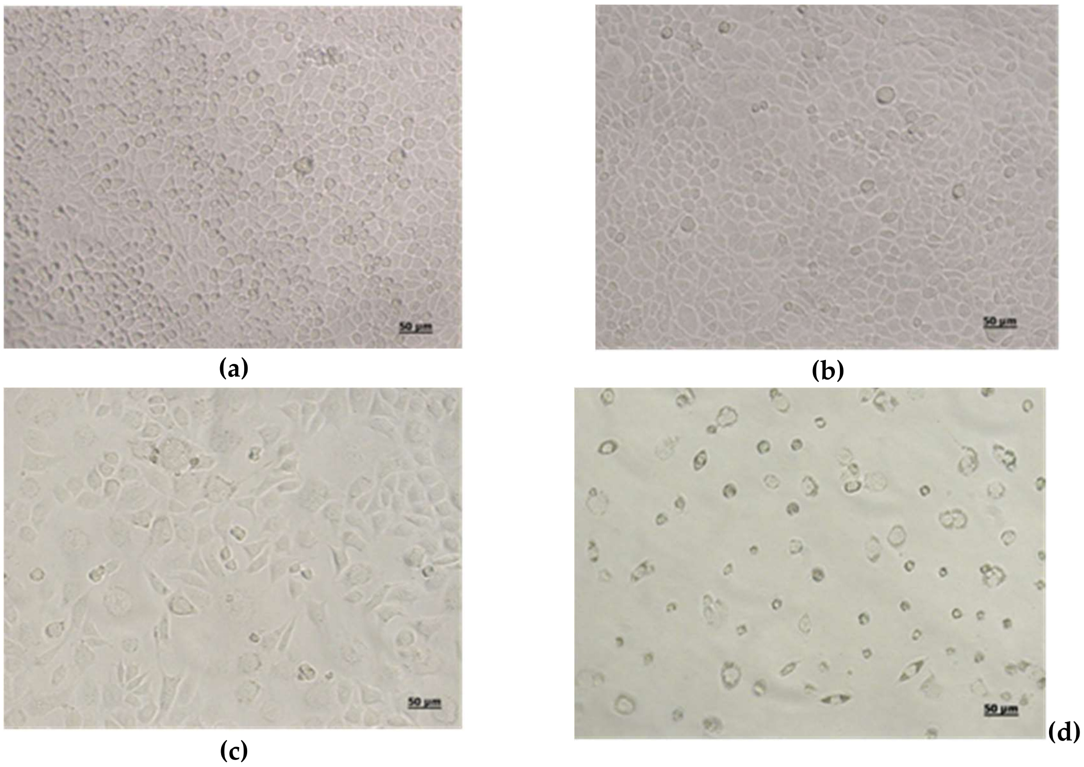

Figure S1: Light micrographs of NCTC clone L929 cells untreated (a) and treated with different concentrations of atomized product: 20 mg/ml (b), 25 mg/ml (c) and 30 mg/ml (d).

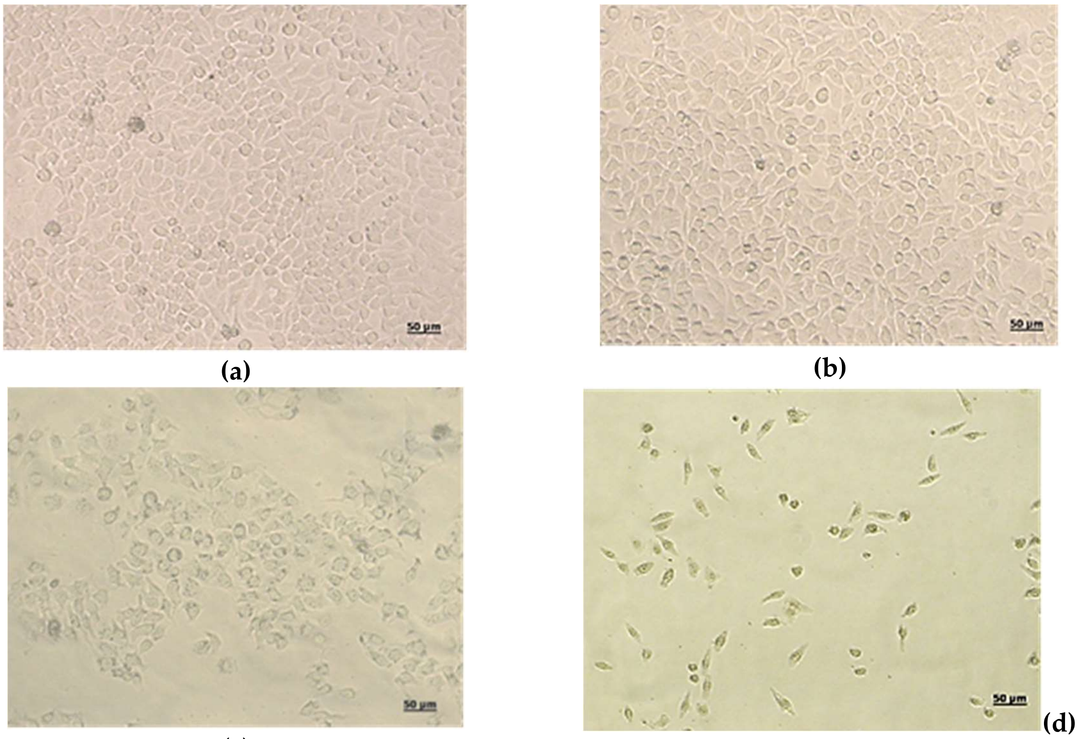

Figure S2. Light micrographs of Hep-2 cells untreated (a) and treated with different concentrations of atomized product: 15 mg/ml (b), 20 mg/ml (c) and 30 mg/ml (d).

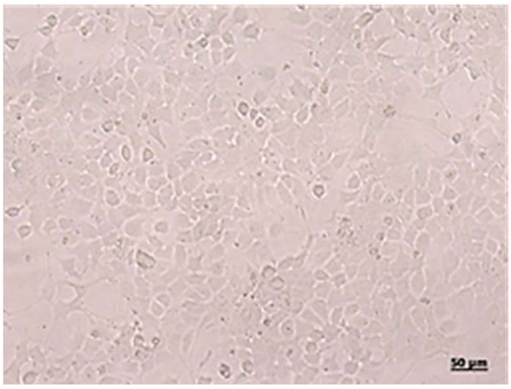

(a)

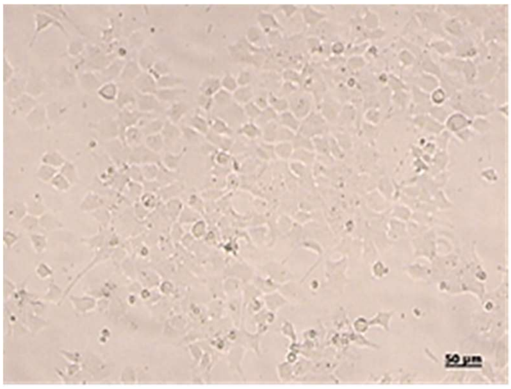

(b)

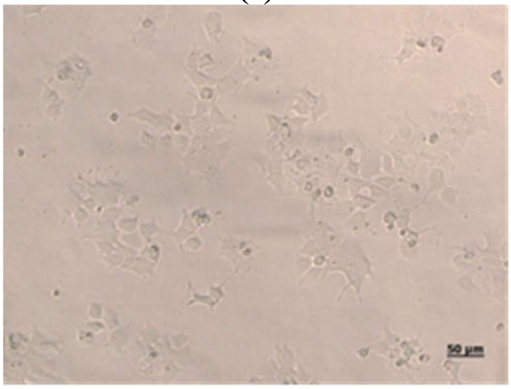

(c)

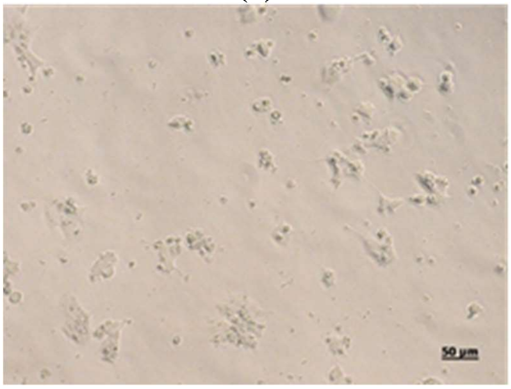

(d)

Figure S3. Light micrographs of Caco-2 cells untreated (a) and treated with different concentrations of atomized product: 10 mg/ml (b), 15 mg/ml (c) and 20 mg/ml (d).
